# Indocyanine Green-Enhanced Colorectal Surgery—between Being Superfluous and Being a Game-Changer

**DOI:** 10.3390/diagnostics10100742

**Published:** 2020-09-24

**Authors:** Catalin Alius, Corneliu Tudor, Cristinel Dumitru Badiu, Ana Maria Dascalu, Catalin Gabriel Smarandache, Alexandru Dan Sabau, Ciprian Tanasescu, Simona Andreea Balasescu, Dragos Serban

**Affiliations:** 1 IVth Surgery Department, Emergency University Hospital Bucharest, 050098 Bucharest, Romania; alius.catalin@gmail.com (C.A.); lulu.tudor@gmail.com (C.T.); catalin.smarandache@umfcd.ro (C.G.S.); sim_deea90@yahoo.com (S.A.B.); 2 Faculty of Medicine, University of Medicine and Pharmacy “Carol Davila”, 020011 Bucharest, Romania; cristian.badiu@umfcd.ro; 3 General Surgery Department, Emergency Clinical Hospital “Bagdasar Arseni”, 041915 Bucharest, Romania; 4 IIIrd Department of Surgery, Faculty of Medicine, Lucian Blaga University of Sibiu, 550169 Sibiu, Romania; alex.sabau@gmail.com (A.D.S.); ciprian.tanasescu@ulbsibiu.ro (C.T.)

**Keywords:** indocyanine green, near-infrared (NIR) fluorescence, colorectal surgery, anastomotic leaks, lymph node mapping

## Abstract

Nowadays, surgical innovations incorporate new technological conquests and must be validated by evidence-based medicine. The use of augmented reality-assisted indocyanine green (ICG) fluorescence has generated a myriad of intraoperative applications such as demonstration of key anatomical landmarks, sentinel lymph nodes, and real-time assessment of local blood flow. This paper presents a systematic review of the clinical evidence regarding the applications of ICG near-infrared (NIR) fluorescence in colorectal surgery. After we removed duplicate publications and screened for eligibility, a total of 36 articles were evaluated: 23 on perfusion assessment, 10 on lymph node mapping, and 3 on intraoperative identification of ureters. Lack of homogenous studies, low statistical power, and confounding evidence were found to be common amongst publications supporting the use of ICG in colorectal surgery, raising concerns over this seductive technique′s cost efficiency and redundancy. The compiled data showed that ICG NIR fluorescence may be a game-changer in particular situations, as proven for low colorectal anastomosis or lateral pelvic lymph node dissection, but it remains controversial for routine use and sentinel lymph node assessment. Further randomized studies are needed to confirm these conclusions. Future research directions include tumor-targeted fluorescence imaging and digital software for quantitative evaluation of fluorescence.

## 1. Introduction

Visionary ideas have almost never been embraced by the academic community without intense scrutiny concerning their scientific principles and favorable peer perception.

Not long ago, the introduction of laparoscopy faced strong opposition and its promotor, Dr. Eric Muhe was ridiculed with derogatory remarks. However, nowadays, minimally invasive procedures are the norm and their benefits are irrefutable [1].

The use of ICG in surgery was envisaged three decades before laparoscopy, but despite being innovative and potentially path-breaking, it did not thrive due to insufficient technological development and lack of researchers in the field [2]. Almost 70 years after Dr. Fox had asked one of his patients for a safe intravenous dye that could be used for functional studies on the heart, today, surgeons are using ICG with increased interest in augmented reality surgery. The molecule of ICG has a unique quantum profile, being able to emit endogenous fluorescence when a beam of near-infrared light is cast upon it. This method allows for the accurate identification of anatomical structures and real-time assessment of local blood flow. It has been used for decades in ophthalmology for imaging retinal and choroid blood flow, due to its ability to provide deeper information about choriocapillaris, beneath the pigmented retinal layers [3,4]. Today there is an exponential growth of papers pertaining to surgical uses of ICG and a myriad of intra-operative and clinical applications demonstrating not only a genuine interest but possibly the much sought-after wide peer acceptance. Enhancement of conventional human senses through augmented reality that can accurately locate anatomical structures and provide real-time functional information on tissues on the basis of ICG’s fluorescent properties is not only seductive but has the potential to be a game-changer in the surgical field. Like all newly developed technologies, near-infrared fluorescence (NIR)-assisted surgery must satisfy unmet clinical needs, be safe and cost-effective, and provide advantages over traditional approaches.

Whether the employment of ICG in surgical procedures is always pertinent, or whether it is contributory to the operative or clinical decision, is yet to be determined. Still, the method has strong scientific support, and evidence-based data suggest its usefulness in specific situations such as identification of anatomical landmarks and real-time assessment of local blood supply. Despite its many theoretical applications, the technique has limitations generated by auto-quenching, diffusion, dimerization, rapid half-life in the bloodstream, and low quantum yields [5,6,7]. The safety profile of the dye is excellent, and its use became very appealing to an increasing number of surgeons who are using it off-label for various surgical applications. Because some of the studies related to the use of ICG in colorectal surgery are pushing to demonstrate the obvious or the unnecessary, we asked ourselves whether ICG is used redundantly in particular circumstances.

This paper offers a critical perspective on the indications and limitations of ICG in colorectal surgery based on previous clinical available data.

## 2. Materials and Methods

We performed a systematic review on articles in English published up to 2020 identified on PubMed, Springer Nature, and Wiley Online Library via specific interrogations such as “indocyanine green” AND/OR “indocyanine green fluorescence” AND (“colorectal” OR “rectal” OR “colon”) AND (“anastomotic leaks” OR “lymph node”). Criteria for inclusion in this systematic review were observational studies and case series of patients with colorectal cancer undergoing open or laparoscopic surgery using indocyanine green NIR for various purposes. Exclusion criteria were studies with no adequately described surgical procedure, as well as studies evaluating types of cancer other than colorectal cancer. Editorials, reviews, case reports, commentaries, letters, and book chapters were not included. 

All papers were categorized on the basis of The Oxford Centre for Evidence-Based Medicine, and two reviewers analyzed the abstracts for inclusion in the systematic review. A Preferred Reporting Items for Systematic Reviews and Meta-Analyses (PRISMA) flow chart was employed to screen papers for eligibility.

## 3. Results

**Figure 1:**
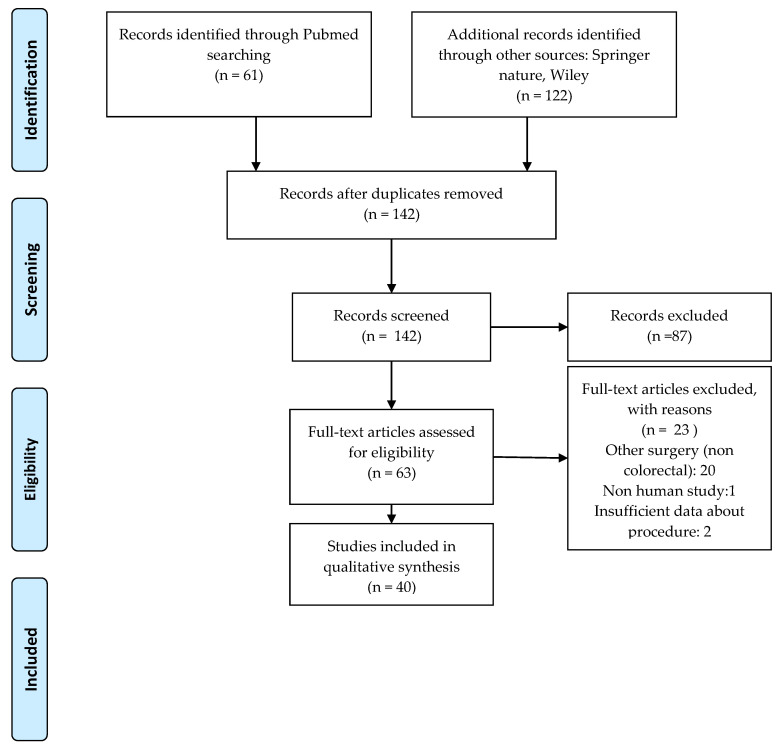
Preferred Reporting Items for Systematic Reviews and Meta-Analyses (PRISMA) flow diagram.

The initial search returned 175 papers published between 2008 and 2020 regarding the use of ICG near-infrared fluorescence (NIR) in digestive surgery in the three databases. After we removed duplicates and screened for eligibility, a total of 36 articles were included in this review. A PRISMA flow diagram is shown in Figure 1.

### 3.1. The Molecule of ICG and the Principle of NIR-Assisted Surgery

The molecule of ICG is a polymethylic cianyne showing both hydrophilic and lipophilic properties due to its sulphate groups and polycyclic rings. The amphiphilic character of the substance makes it soluble in aqueous solutions and suitable for intravenous use [6,7,8]. It binds with circulating albumins and lipoproteins and is excreted into the bile almost unchanged after hepatic extraction. Its half-time in the blood stream is between 3 to 5 min [9]. Indocyanine green is very sensitive to ultraviolet light, and therefore it is commercialized as a lyophilisate and kept in dark vials and should be used at maximum 6 h after reconstitution with sterile water. It is crystallized using iodized salts, which makes it contraindicated in patients with iodine allergies.

Although all members of the cyanine family exhibit fluorescent properties it is the spectrum of its emission light that makes ICG a uniquely suited dye for intracorporeal use. Different from any other fluorophores, with an excitation spectrum around 750nm and an emission spectrum peaking at around 810–830 nm, this molecule fluoresces in NIR [6,7,8]. Hence, it conveniently avoids endogenous interferences produced by water and tissue proteins, a very common drawback with other dyes (methylene blue, isosulfan blue, or patent dye blue), which emit in the visible spectrum. Another advantage of ICG is the excellent safety profile, as opposed to anaphylactic reaction concerns regarding the use of isosulfan blue or patent dye blue, or the local toxicity previously reported for methylene blue, leading to inflammation, blue discoloration, and skin and fat necrosis [8].

In abdominal surgery, ICG fluorescence can be achieved either in open surgery, by using infrared vision cameras, but with the discomfort that it cannot be overlapped over the real-light image, or, more comfortably, in laparoscopic and robotic surgery, where additional software display the fluorescence overlapped with a conventional image, resulting in real assistance during surgery.

Multiple uses of ICG have been reported over the past 20 years in various surgical subdomains, but there are no consensual recommendations regarding the indications and protocols of using ICG in daily surgical practice. In colorectal surgery, the most documented use of NIR fluorescence was in evaluation of the blood supply in colorectal anastomosis in order to prevent anastomotic leaks (23 studies), followed by assessment of lymph nodes during dissection in colorectal cancer surgery (17 studies). Recently, other uses of ICG are emerging, but there is insufficient clinical evidence to be included in the review: detection of the peritoneal metastasis, as well as identification of the ureters during extensive dissection in colorectal oncological surgery [9,10].

### 3.2. Impact of ICG-Enhanced Surgery on Anastomotic Leak Rates

#### 3.2.1. The Anastomotic Leaks (AL)—A Significant Factor of Outcome in Colorectal Surgery

Colorectal cancer is the second most common cancer in women and the third most common in men, generating an increase in the number of operations and subsequent complications such as anastomotic leaks (AL) [11,12]. Authors report average leak rates ranging from 1 to 3% for ileocolic anastomoses and up to 10–20% for low colorectal anastomoses.

While relatively uncommon in colorectal surgery, AL adversely impact postoperative outcomes worldwide, being one of the most frequent contributors to all 30-day mortality and a cause for long-term morbidity including poor bowel function, reduced quality of life (QoL), increased risk of cancer recurrence, and high rates of permanent stoma [13,14].

Pathogenesis of anastomotic leaks is multifactorial involving both patient- and surgical technique-related factors [15]. Patients with no Riolan–Haller arch will only rely on the internal iliac artery retrograde flow through the middle rectal arteries to feed a bowel anastomosis in cases of a “high ligation” left hemicolectomy. Those with a poorly developed marginal arcade with anatomical variations and those in whom a long sigmoid stump is left will also have an increased risk of anastomotic complications [16]. Fluorescent assessment with ICG could demonstrate the blood supply of the colon and provide real-time feedback on the perfusion of the proximal and distal anastomotic limbs.

#### 3.2.2. Study Designs—Comparative Aspects

Our research identified 71 articles related to the use of ICG in vascular assessment of anastomoses, but only 23 papers remained eligible for review after a PRISMA evaluation [13,17,18,19,20,21,22,23,24,25,26,27,28,29,30,31,32,33,34,35,36,37,38]. The ensuing comments and critical reflections are based on data from 3146 patients enrolled in 23 non-randomized studies on the use of ICG-enhanced NIR fluorescence for vascular evaluation of anastomoses in colorectal surgery involving open, laparoscopic, or robotic procedures (Table 1).

All studies included in the review aimed to assess the utility of ICG NIR-enhanced surgery in correct estimation of the resection tranches to ensure a sufficient blood supply in order to prevent AL. The final outcomes were the incidence of AL in the ICG group and, secondly, to what extent NIR fluorescence changed the initial surgical decision, and therefore what should or should not be introduced in current surgical practice. There are significant differences, which made a meta-analysis unreliable, due to multiple sources of bias: the study design, patients’ selection, dose of ICG, evaluation of fluorescence, and criteria used for evaluation of outcomes.

The sample size varied from 18 [21] to 504 patients [12]. Some authors included all colorectal surgeries [13,17,25,29,32,34,35,36] for both benign and malign pathologies, as well as for all locations, while others focused on the most vulnerable colorectal segments with regard to blood supply, namely, the left colon and low colorectal anastomosis [19,20,21,22,23,24,26,27,28,30,31,32,33,37,38]. In 13 of the studies [12,15,17,18,19,21,22,23,24,29,30,33,34], only the group of patients receiving ICG was described, while in 10 studies [16,20,25,26,27,28,31,32,35,36], comparative lots of ICG and non-ICG patients were analyzed to evaluate the final outcome. In many cases, however, the control lots were retrospective, chosen from patients attending the same center in previous years. The diagnostic of AL was clinical, in most cases, while Sherwinter et al. and Ohya et al. defined it more accurately [17,19] by computer tomomography (CT) exam, also considering minor AL, with subclinical findings, but which can be a cause of long-term morbidity.

#### 3.2.3. ICG NIR Fluorescence Assessment: Doses, Devices, Protocols

In all studies, the ICG angiography was performed by slowly intravenous injection of a ICG solution of 2.5 mg/mL, but various doses were used (0.1–0.3 mg/kg or standard doses of 3.75–10 mg). No safety incident was reported. The colon perfusion was assessed in the following 2–5 min.

While most of the authors employed a trans-abdominal approach using the integrated NIR function of the optical system used in laparoscopic/robotic surgery (IMAGE1 S, Karl Storz; PINPOINT, Stryker/Novadaq; FIREFLY, da Vinci, etc.), Sherwinter et al. [17] proposed a trans-anal endoscopic evaluation that allowed for simultaneous leak testing and perfusion assessment. Other authors opted for extereriorization of the resection tranches and examination outside peritoneal cavity, with a PDE-neo system. There is no consensus regarding the intensity of fluorescence that would be considered “at risk”, with this being a subjective evaluation by comparison with the adjacent segments. Son et al. attempted to determine quantitative benchmarks in terms of the maximum level of fluorescence and the time during it is reached, defining a satisfactory blood supply as where the time ratio (TR = T1/2MAX/TMAX) was below 0.6 s [31].

Some studies also considered a second bolus of ICG after anastomosis was performed, in order to confirm a good perfusion or reshape it accordingly [13,23,25,26,27,28,29].

#### 3.2.4. The Rate of AL with ICG NIR-Enhanced Surgery

In a critical analysis of available data, we found significant different outcomes reported by studies that enrolled all colorectal surgeries, as opposed to studies that analyzed a more homogenous group of left colon or colorectal surgeries.

A change in surgical decision prompted by NIR evaluation (including additional resections or repositioning of the transection lines towards segments with richer blood supply) was encountered in 5.68% (1.6–16.7%) of patients, when study groups included all colorectal surgeries, and of 25.74% (5.7–88%) when only patients with left colorectal cancers were included. 

The average rate of AL reported after ICG NIR fluorescence surgery was of 2.11% in studies that included all colorectal surgeries, with variations between 0 and 7.5%. Only three studies compared this outcome with a control group, and each encountered different results, but all without statistical significance. Su et al. [29] found no AL in both groups and Tsang et al. [34] found a slightly decreased incidence when ICG fluorescence was used. Only one study [18] reported a slightly higher incidence in the ICG group, but the control lot was chosen retrospectively from previous data, raising a very high concern of selection bias.

When only left colo-rectal surgery was evaluated, the average rate of AL in ICG group was of 3.75% (0–8.8%), as opposed to an average of 11.45% (5.4–14.7%) in the non-ICG group. When a control group was used, a significant lower number of AL when ICG was used to assess the blood supply at the anastomotic level was encountered in all studies [23,24,26,27,28,30,33,37]. In these groups, anatomically predisposed to high risk of vascular insufficiency, the incidence of AL after evaluation by ICG dropped below 6%, compared to the estimate rate of 10–15% in the current literature. The authors appreciate that the lower the level of the anastomosis, the higher the risk of performing a poorly vascularized anastomosis, emphasizing the importance of intraoperative fluorescence in changing the surgical decision.

### 3.3. The Usefulness of ICG in Sentinel Lymph Node (SLN) and Lymphatic Basin Identification

Compared to traditional dyes, ICG offers undeniable advantages in assessing the lymphatic flow; it allows for greater accuracy than Lymphazurin, and methylene blue in detection of deeper lymphatic structures. This aspect is particularly important in obese patients, making the dissection easier and less extensive. When compared to radioactive dyes such as 99 m Tc, ICG is cheaper and avoids the risks related to radiation exposure [39,40].

#### 3.3.1. ICG NIR Fluorescence in Identifying SLN in Colorectal Cancer Surgery

In our research, we analyzed 12 [35,40,41,42,43,44,45,46,47,48,49,50] clinical studies regarding the role of ICG NIR in identifying the sentinel lymph node (SLN) in colon cancer surgery, in terms of procedure, detection rate, and sensitivity of the method. Overall, 337 patients were enrolled. The results are presented in Table 2.

##### Study Designs—Comparative Aspects

Overall, 337 patients with colon cancer, preoperatory classified as stage I or II, were enrolled. Preoperatory lymph node metastasis on CT or magnetic resonance imaging (MRI) exam were exclusion criteria in all studies, except that of Nishigori et al. [35]. Regarding the tumoral stage, Curie et al. and Cahill et al. [42,49] considered only T1-2, while others did not take into account the tumoral extension; meanwhile, there was no preoperatory lymph node metastasis detected [40,42,43,44].

The outcomes were on one hand the detection rate of SLN, after ICG injection, and on the other hand the specificity, sensitivity, and negative predictive value of SLN detected by ICG fluorescence, after histopathological examination of all lymph nodes in the lymphatic basin, removed along with the tumor. In some studies, histopathological assessment was by hematoxylin–eosin examination only [35,43,44,45,47,48,49], while in others, ultrastaging of SLN by immunohistochemistry was also included [40,41,42,46,50]. A particular study design was that of Watanabe [45], who focused on detecting the aberrant lymphatic flow, which may be present in splenic flexure cancers.

##### Technical Aspects of ICG Injection: Dose, Concentration, Technique

The concentrations of ICG used in reviewed studies ranged from 0.5 to 5 mg/mL and the doses from 0.5 to 5 mL, although the overall results were similar. The ICG was administrated either subserously, by open or laparoscopic approaches, or submucosaly, by the endoscopic approach, in two or four peritumoral points. In one study, the ICG was administrated intravenously [43]. When ICG was injected around the lesion of interest by laparoscopic approach, there was an increased risk of accidental spillage and fluorescent contamination of the optical field. This might render the procedure of SLB or nodal mapping futile and time consuming, hence the preference of some authors for endoscopic delivery of the dye [46].

The time elapsed between ICG injection and detection of fluorescent lymph nodes by NIR light ranged between 5 and 30 min. Some authors argue that the use of less concentrated solutions of ICG will diffuse quicker into the surrounding tissues. Andersen et al. and van Der Pas et al. preferred a mix of ICG with albumin to prevent dispersal; however, they did not report superior results in terms of lymph node identification [46,50].

The images acquisition was made either ex vivo, after specimen removal [43,44], either in vivo, by NIR function of laparoscopic system or in open surgery, by additional use of an NIR lamp.

##### Outcomes: Overall Sensitivity and Specificity of ICG Near-Infrared Fluorescence

ICG proved to be useful in assessing lymph flow and locating SLN in colorectal cancer, with a detection rate of up to 90–95%. Yet, clinical studies showed a variable sensitivity (ranging from 0% to 85.6%) in detection of nodal metastases following histopathological examination and ultra-staging of the harvested specimens of SL. These differences can be explained by variable methods used, but also by the heterogeneity of the study groups, as tumor staging. Van der Pas et al. [50] communicated 0% sensitivity, which can be related to defective technique, as also was noticed by Liberale et al. [51].

Carrara et al. raise the possibility of ultraconservative colonic resection in low stages of colorectal cancers on the basis of the negative predictive value of the sentinel lymph node (SL) [40]. The authors suggest harvesting the lymph node/nodes after peritumoral injection of ICG followed by pathology assessment with one-step nucleic acid amplification technique (OSNA). This study demonstrated that SL was found at around 4 min in 92 out of 95 patients and the negative predictive value of the test was 96.2%. Furthermore, 50% of the SL were located in the pericolic basin and 7.3% within the central nodes. However, this is enough to validate the technique as the sensitivity was limited (73%), meaning that in 37% of cases there were metastatic lymph nodes detected other than SLN.

Curie et al. consider that many previous studies on SL in colonic cancers had been compromised by the use of ineffective dyes and inclusion of patients with advanced disease, and that with adequately selected cases (T1/T2), the technique utilizing ICG could improve surgical outcomes and limit extensive resections [52], while Andersen raises the question of micro-metastases undetected by sentinel lymph nodes and under-staging in certain patients [42].

Watanabe et al. propose the use of ICG to study the path of lymph flow in real time, considering this technique for sampling lymph nodes in the pelvis to be superior to ultra-staging (serial sectioning and use of immunohistochemistry) of the sentinel node [45].

#### 3.3.2. ICG NIR Fluorescence in Lateral Pelvic Lymph Node Dissection (LPLD) in Rectal Cancer

Lateral pelvic lymph node dissection is an invasive surgical procedure in rectal cancers with MRI documented/suspected metastasis in internal iliac and/or obturatory lymph nodes, and it is associated with an increased an risk of associated genito-urinary morbidities due to extensive dissection required in the extraperitoneal space. There are few papers that document the role of ICG in LPLD. The first to investigate the lateral pelvic lymph flow in rectal cancer by the use of ICG NIR fluorescence was Kawahara et al. in 2007 [53]. The authors stated that, if present (43%), lateral pelvic wall lymph node drainage was limited exclusively to the peri-internal iliac artery nodes, with none in the obturatory lymph nodes. The first report of a lateral region SLN in lower rectal cancer guided by ICG by means of a near-infrared camera system was of Noura et al. [54] on a group of 25 patients. He establishes the optimal concentration and dose for lateral pelvic lymph node assessment at 1 mL 5 mg/mL solution, proving that lymph nodes are not visible at lower concentrations. Alternatively, injection of 2 mL of hyperfluorescent ICG will cause rapid diffusion into the surrounding fatty tissue [54]. The study found a detection rate of 92% and a sensitivity of 82% in detecting SLN on the lateral pelvic wall. A limitation of the study is that LPLD was performed in all cases when SLN was positive (three patients), but only in three out of 20 cases with negative SLN, and thus sensitivity was actually calculated on a much smaller group (six patients).

In a case series of five patients, Handgraaf et al. [55] evidenced the utility of ICG NIR fluorescence in identifying rectal tumors during laparoscopy (in 80% of cases) when previously submucosally and endoscopically injected. As a plus, with comparison to ink tattooing, ICG also follows the lymphatic flow and evidences the SLN.

Zhou et al. and Boni et al. [56,57] showed the usefulness of ICG in harvesting pelvic lymph nodes during lateral pelvic lymph node dissection, demonstrating that the ease of localization with the help of augmented reality, especially in obese patients, allows for collection of a large number of lymph nodes (11.5 ± 5.9 vs. 7.1 ± 4.8, *p* = 0.017), with less blood loss (55.8 ± 37.5 mL vs. 108.0 ± 52.7 mL, *p* = 0.003) and over a shorter period time compared to traditional surgery [56]. In these studies, the indication of LPLD was established before surgery, not by ICG assessment of SLN on the lateral pelvic wall. 

## 4. Discussion

Despite a plethora of publications on the use of ICG in colorectal surgery, there is high heterogeneity in terms of study designs and very few systematic reviews addressing the use of ICG from a multi-centric perspective, hence the difficulty and high risks of biases in meta-analyses. All the studies reviewed in this paper were non-randomized and showed variability in selection of patients, doses, designs, and final outcome definitions.

### 4.1. ICG NIR-Enhanced Surgery in Preventing AL: When Should Be It Performed by Routine?

One of the shortages encountered in the reviewed studies is that the assessment of hypo-fluorescence is subjective, judged by each surgeon by comparison with adjacent colic segments. The adequacy of the transection line as determined by ICG fluorescence was interpreted subjectively and could be seen as a limitation. One aspect requiring reflection is that NIR fluorescence depends on the distance between the camera and the examined structure. Therefore, an anastomosis with incipient or minute vascular problems could show good fluorescence if examined from too short a distance or, on the contrary, a healthy piece of bowel might display weaker fluorescence if the device is held too far away from the area of interest. In the evaluated studies, the fluorescence of the anastomosis was assessed qualitatively in relation to the fluorescence of the adjacent colic segments, and thus keeping the same distance between the camera and the examined digestive segment is important for uniform results. The minimal blood supply requiring a viable anastomosis in currently unquantifiable by neither eco-Doppler assessment nor ICG fluorescence.

Although the blood supply is the dominant factor determining AL rates, one must consider the multifactorial etiology of this complication. In the reviewed studies, AL due to other causes (mechanical, stapler related, vicinity to uretero-neocystostomy fistula) were also included in the final outcome evaluation [25,32,36].

Fluorescent enhanced surgery is promising, and all evidence points towards a safe, cost-effective, and easily reproducible technique. No adverse effects, either local or systemic, were reported in the reviewed studies.

Related to the impact of ICG on the reduction of AL, of the total number of papers reviewed, none concluded that ICG is detrimental, and they outlined that it does not improve short-time outcomes. Still, some had various biases or showed insufficient statistical power. However, the overall perception is that ICG does improve the rates of anastomotic complications, especially in left colorectal surgery, with solid randomized controlled trials being expected to confirm this [58,59,60,61].

Ohya et al. analyzed fluorescent abnormalities in end-to-end anastomoses performed in 400 patients with colon cancer, distinguishing three degrees of fluorescence around anastomoses: (i) good fluorescence within 60 s from ICG injection, (ii) delayed fluorescence at over 60 s following the injection, and (iii) non-fluorescence in cases with a minimal fluorescent signal. In 11 patients (2.8%), macroscopically normal anastomoses showed delayed (three patients) or non-fluorescence (nine patients), prompting refashioning. The overall AL percentage was 1%, but the study group had a large proportion of right-sided colorectal cancers (58%) requiring ileo-transverse anastomoses, which are less prone to fistulous complications [17].

Similar results are encountered in studies on patients with all types of colorectal cancers requiring right hemicolectomies. The influence of ICG NIR fluorescence on changing the surgical decisions in ileocolic anastomoses is limited, and hence its routine use is controversial at least. [17,25,29,32,34,36]. One intuitive explanation is that the overall risk of AL in right bowel surgery is low due to adequate blood supply of the ileum and the right colon.

Somashekhar et al. reported in 2020 a series of 50 patients within whom the transection line established conventionally by means of subjective evaluation was reassessed with ICG. In 88% of the cases, the second look prompted a change in the surgical decision. Although none of the patients in whom the anastomosis site was reconsidered developed leaks, for the vast majority of the cases, the authors fashioned protective ileostomies [21].

Arezzo et al. searched PubMed, Embase, Cochrane Library, ClinicalTrial.gov, EU Clinical trials, and ISRCTN in 2019 in order to analyze the assessment of anastomotic vascularization in rectal cancer surgery by intraoperative use of ICG compared with standard practice. After identifying 947 papers, only nine were finally included in the systematic review. A total of 862 patients were assessed with ICG and 468 were assessed by conventional means. Incidence of AL at 30 days was 4.2% in the ICG group and 11.3% in the control group. The authors compared relatively homogenous populations in terms of age, co-morbidities, gender, and body mass index (BMI) [62].

In the present review, we demonstrated that although ICG NIR fluorescence might not be recommended as a routine examination in colonic surgery, it has been proved to be useful in vascular assessment of anastomoses in left colic and rectal cancer surgery. The lower the anastomosis level, the more important the assessment of the blood supply in preventing future AL. Further randomized studies are expected to determine the adequate doses and quantitative parameters to define a satisfactory blood supply.

Creating more elaborated surgical techniques usually prolongs surgery, but the intraoperative fluorescent angiography with ICG takes only 4 to 5 min and is very safe. With the development of novel technologies, NIR fluorescence is practically an option included in most of the laparoscopic and robotic image acquisition systems, with the possibility of overlapping infrared and visible spectrum images in real time. The ICG fluorescence-based enhanced reality has passed the experimental stage and became useful in everyday surgical practice. At the expense of a few additional minutes on the operative times, all authors reported that the final outcome was in all cases a decreased incidence of the AL. One should bear in mind that the definitions of this complication varied widely from suggestive clinical criteria to CT documentation of perianastomotic collections or peritonitis. However, more randomized clinical studies on a higher number of patients are needed to confirm the most adequate dose and to develop high-contrast, quantitative algorithms for blood supply evaluation.

### 4.2. ICG NIR Enhanced Surgery in SLN: Is It a Game-Changer?

#### 4.2.1. Controversies in the Concept of SLN in Colorectal Cancer

Increasing adherence to radical oncological principles such as complete mesocolic excision/D3 colonic resections have improved survival in patients with colorectal cancers, but up to 20% of the cases with early colonic cancer and pN0 will develop metastases within five years following surgery [62,63,64,65,66]. This suggests that a certain number of patients with lymph node metastasis at the initial surgery remain undetected. In the most widely accepted surgical protocols, the tumor status of the SLN does not change the extent of resection because en bloc resection of the primary colorectal cancer includes regional lymph nodes, but the results of SLN-based nodal ultrastaging can improve identification of candidates for adjuvant therapy of CC, a treatment that is highly effective for metastatic disease but too toxic and expensive for routine use in stage I–II with negative lymph nodes [67]. Using ICG for lymphatic mapping could allow full harvesting of nodal basins with ease.

On the other hand, in early colonic cancer, sentinel lymph node assessment could lead to limited resections with functional benefits in the long term [40]. However, the real problem of SLN biopsy in colorectal cancer is the procedure sensitivity rate, which remains relatively low [67]. The main factors that can be incriminated are the selection of patients, the surgeon’s experience, the dye used, the different surgical techniques, and histopathological assessment of the specimens. Most authors do not recommend this technique in cases with lymph node metastasis present on preoperatory CT/IRM exam. Regarding the tumor staging, the opinions differ—while Carrara et al., Hirsche et al., and Liberale et al. do not take into account the tumoral invasion in the colic wall [40,41,43,44], Cahill et al. and Curie et al. consider that only T1-2 should be considered for limited resections on the basis of SLN because of possible impact of tumoral development upon the pre-existent lymph flow and aberrant lymphatic drainage because of occlusion of the lymph channel by the tumor [42,49].

#### 4.2.2. ICG NIR-Assisted Surgery Performance in Detecting SLN

ICG NIR fluorescence proved to be a reliable tool in evaluation the lymphatic flow and in identifying the SL. However, with a universally accepted lower clinical significance for the concept of SL in colorectal surgery in comparison to breast and skin malignancies, the use of this method in establishing suitable candidates for limited colonic resections remains controversial [60,62,63,64,68].

In a systematic review and meta-analysis performed by Emile et al. in 2017 on 12 studies, preoperative injection of ICG had the highest sensitivity (100%) and intraoperative injection had the highest specificity (100%) and accuracy (80.2%) rates in detecting SLN. The percent of patients with early stage CRC had an impact on the outcome of NIR fluorescence. When the percent of patients with early stage tumors was >50% of the sample size, the median sensitivity, specificity, and accuracy were all much better than in studies with an increased percentage of advanced cancers (all 100% vs. 76%, 87.2%, and 68.8%, respectively) [69]. The heterogeneity among studies regarding dosage and administration protocol may be also a cause for the increased variability in final outcomes.

Liberale et al., in a systematic review in 2018, found that ICG fluorescence-enhanced surgery is a safe procedure, with a specificity of 65–100% and an extremely variable sensitivity between 0–100% for colon cancer among the evaluated studies. The lowest sensitivity was encountered in the study of van Der Pas et al., who used a ICG and albumin solution. Another limiting factor is the different percentage of early and advanced colorectal cancer included in various studies. Early cancer patients are better candidates for SLN-assessing techniques [70].

Villegas-Tovar et al. published a systematic review on 11 studies and concluded that the use of ICG for detection of SL is superior in laparoscopic procedures, but has an overall poor performance [71]. In all these studies, the sensitivity of 64.3% (51–76%) and specificity of 65% (36–85%) were low, but the pathology analysis was performed by conventional examination using hematoxylin–eosin (HE). With OSNA, the numbers would probably improve towards a higher acceptance of the technique. There is a higher hesitation to incorporate it in the standard practice due to non-convincing evidence of improved outcomes compared with traditional techniques. These are supported by oncological radicality principles taught and popularized by our mentors and strongly embedded in surgical training programs. Changing paradigms to ultra-selective colectomies and introduction of SLB to the daily routine in colorectal surgery must be based on very well documented studies with robust designs and a large number of participants. We believe that, to date, the use of ICG-enhanced surgery has failed to emerge as a game-changer and that the fundamental question regarding its superfluousness remains unanswered and is an invitation to further scrutiny and research.

Future perspectives will involve tumor-targeted fluorescence imaging by using a fluorescent dye coupled with a specific antibody targeting a tumoral antigen (e.g., ACE) in order to identify the tumor limits and the metastatic disease along the lymph node basin [72,73].

#### 4.2.3. ICG NIR-Assisted Surgery in LPLD

Up until now, there has been no consensus regarding the use of ICG for lymphatic mapping or SLB, but it has been proven to be useful in the assessment of the lymphatic flow in particular circumstances and it can be a helpful tool during LPLD in rectal cancer surgery. A more targeted dissection brings the benefits of minimal blood loss and reduced damage in the adjacent tissue and can shorten the learning curve for this technique [56,57].

### 4.3. A Step Forward from Superfluous: Attempts in Standardization and Quantification of ICG NIR-Enhanced Surgery

The reviewed data show that a more systematic approach in selection of patients and techniques are needed. There is an emerging preoccupation to create quantitative, objective parameters to help surgeons evaluate the NIR fluorescence and reach comparable results [74,75,76,77,78]. Quantitative ability of a fluorescence image system will play a crucial role in its widely intraoperative use. A step forward was the introduction of a computer-assisted method for evaluating the fluorescent signal described by Diana et al., who proposed superimposing perfusion cartography onto real-time laparoscopic images to create the so-called fluorescence-based enhanced reality (FLER) [77].

Fluorescence-based enhanced reality FLER allows imaging of the quantified fluorescence signal and provides a reproducible estimation of bowel perfusion. The clinical validation of these new tools will come in following years.

## 5. Conclusions

Near-infrared (NIR)-enhanced surgery has the potential to change paradigms, but up until now, there have been no guidelines or standardized uses for this method. Promising studies and favorable systematic reviews do not suffice when it comes to embracing a new surgical technique. Although the safety profile of the ICG is unquestionable, the sometimes-excessive use of the method would only increase costs and the complexity of an operation. Lack of homogenous studies, low statistical power, and confounding evidence is common amongst publications supporting the use of ICG in colorectal surgery. Viewed by some authors with skepticism, by others with enthusiasm, NIR augmented reality seems to be shaping its place in colorectal surgery. Adding NIR to the image acquisition software in laparoscopic and robotic surgery facilitates the use of this augmented reality when the surgeon needs more real-time information about the blood or lymphatic flow or the precise location of anatomical landmarks in order to avoid iatrogenic damage. Although superfluous if performed by routine in colorectal surgery, ICG NIR may be a game-changer in particular situations, as proven in low colorectal anastomosis or lateral pelvic lymph node dissection.

## Figures and Tables

**Table 1:** Clinical studies regarding the role of near-infrared (NIR) indocyanine green (ICG) in preventing anastomotic leaks (AL) post-colorectal surgery; the first nine studies [13,17,25,29,32,34,35,36] refer to the use of ICG in all colorectal diseases; the following 14 refer to left colon and rectal surgeries only [19,20,21,22,23,24,26,27,28,30,31,33,37,38].

Study, Year	No. Cases (NIR; Control)	Pathology	Type of Surgery	Fluorescence Assessment	Dose	Decision Change	AL% (NIR; Control)	Image Acquisition Device
Ris, F.; et al. [13], 2018	504 NIR only	B/M colorectal diseases (all locations)	Laparoscopy/open/conversion colorectal surgery	Intraperitoneal	7.5 mL before and after	5.8%	2.4% (none in those with revised surgery)	PINPOINT, Stryker
Ohya, H.; et al. [17], 2020	*n* = 400 NIR only	Colon/appendiceal cancer (all locations)	Laparoscopic colectomies	Intraperitoneal	0.25 mg/kg	2.8% (mostly transverse and left colon anastomosis)	1%	IMAGE1 S, Karl Storz PINPOINT, Stryker
Kin, C.; et al. [18], 2015	346 (173;173 historically matched)	M/B colorectal diseases (all locations)	Laparoscopic colorectal surgery	Intraperitoneal	No info	5%	7.5% 6.4%	No info
Boni, L.; et al. [25], 2017	*n* = 107, NIR only	Colorectal cancer, all locations	Laparoscopic right/left hemicolectomy, AR	Intraperitoneal	0.2 mg/kg before and after	3.8%	0.9% (due to stapler related deficiency)	IMAGE1 S, Karl Storz
Su, H.; et al. [29], 2020	189 (84;105)	Colon cancer, >30 cm anal verge	Totally laparoscopic colic resection	Intraperitoneal	Before and after 7.5 mg (3 mL)	4.8%	0% 0% 0% stricture	opto-cam 2100 (Optomedic, Guangdong, China)
Ogino, T.; et al. [32], 2018	74, NIR only	B/M colorectal diseases (all locations)	Laparoscopy/open colorectal surgery	Extraperitoneal	5 mg before	8.1%	1.4% (1 case, not related to ischemia)	Photodynamic Eye System, Hamamatsu Photonics
Tsang, Y.; et al. [34], 2020	131 (63;68)	B/M colorectal diseases (all locations)	Laparoscopic/robotic colorectal surgery	Intraperitoneal	10 mg before	1.6%	3.23% 4.35%	Firefly, da Vinci Xi Olympus OTV-S300 CLV-S200-IR (Olympus, Tokyo, Japan)
Nishigori, N.; et al. [35], 2016	21 NIR only	Colorectal cancer (all locations)	Laparoscopic colorectal surgery	Intraperitoneal	1–3 mL (2.5 mg/mL)	16.7%	0%	D-Light P System/Image 1 SPIES, Karl Stortz; infrared endoscopic camera system, Olympus (Tokyo, Japan)
Santi, C.; et al. [36], 2019	38, NIR only	Colorectal cancer, all locations	Laparoscopic colic resection	Intraperitoneal	5 mL (0,3 mg/kg) before	2.6%	2.6% (mechanical cause)	IMAGE1S, Karl Storz
Sherwinter, D.A.; et al. [19], 2012	*n* = 20 NIR only	B/M Rectal diseases	Laparoscopic LAR	Endoscopy, transanal	1 mL 2.5 mg	N/A 4 cases with hipofluoresce—decision not changed	10% (managed conservatory)—CT diagnosed	PINPOINT, Novadaq (Canada)
Kawada, K.; et al. [20], 2016	*n* = 68 NIR only	Left colorectal cancer	Laparoscopic colorectal surgery	Specimen extracted by the umbilical trocar and examined	5 mg	26.5%	4.5% (symptomatic)	PDE-neo system, Hamamatsu Photonics
Somerskhar, J.P.; et al. [21], 2020	*n* = 50 NIR only	Colorectal cancer	Robotic Sigmoid colectomy, LAR +ileostomy	Intraperitoneal	3 mL, 2.5 mg/mL	88%	0%	FIREFLY, da Vinci
Jafari, M.D.; et al. [22], 2013	*n* = 38 (16;22)	Rectal cancer	Robotic-assisted AR	Intraperitoneal	6–8 mg before	18.75%	6% 18%	FIREFLY, da Vinci
Grone, J.; et al. [23], 2015	*n* = 18, ICG only	Rectal cancer	Laparoscopic AR (1 conversion)	Intraperitoneal	10 mg before and after	27.8%	6% (compared to 15% historically, same center)	PINPOINT, Novadaq
Hellan, M.; et al. [24], 2014	*n* = 40, ICG only	Left colorectal cancer	Robotic-assisted left colorectal surgery	Intraperitoneal	10 mg ICG	40%	5% (with revised anastomosis)	FIREFLY, da Vinci
Jafari, M.D.; et al. [26], 2014	*n* = 139, NIR only	Left colorectal cancer	Laparoscopy-assisted left colectomy, AR	Intraperitoneal	3.75-7.5 mg before and after	8%	1.4% (no AL in cases with changed decision)	PINPOINT, Novadaq
De Nardi, P.; et al. [27], 2020	*n* = 240 (118;122)	Left colorectal B/M diseases	Laparoscopic AR, LAR, left colectomy	Intraperitoneal	0.3 mg/kg before and after	11%	5% 9%	IMAGE1 S, Karl Storz
Kim, J.C.; et al. [28]. 2016	426 (123; 313)	Rectal cancer	Robotic-assisted sphincter-saving operations	Intraperitoneal	10 mg (before+/- after)	Not applicable (site chosen by NIR)	0.8% 5.4%	FIREFLY, da Vinci
Mizrahi, I.; et al. [30], 2018	60 (30;30)	Low rectal cancer, <5 cm anal verge	Laparoscopic LAR	Intraperitoneal	0.1–0.3 mg/kg	13.3%	0% 6.7%	PINPOINT, Novadaq
Son, G.M.; et al. [31], 2019	86, NIR only	Colorectal cancer	Laparoscopic AR (55) Laparoscopic LAR (31)	Intraperitoneal	0.25 mg/kg before	Not applicable	7% good correlation with poor perfusion (TR >0.6)	IMAGE1 S, Karl Storz
Hasegawa, S.; et al. [33], 2020	852 (143;709)	Rectal cancer	Laparoscopic LAR, ISR	Intraperitoneal	5 mg before	17.0%	2.8% 12.4%	IMAGE1 S, Karl Storz; 1588 AIM and SPY (Stryker), HyperEye (Mizuho Medical)
Watanabe, J.; et al. [37], 2020	550 (236;314)	Low rectal cancer	Laparoscopic LAR	Intraperitoneal	0.25mg/kg	5.7%	4.7% 10.4%	D-Light P, Karl Storz; 1588 AIM Platform, Stryker
Wada, T.; et al. [38], 2019	149 (48;101)	Low rectal cancer	Laparoscopic LAR	Specimen exteriorized by the umbilical trocar and examined	5 mg before	27.1%	8.8% 14.7%	PDE-neo system, Hamamatsu Photonics

**Table 2:** Studies regarding NIR ICG detection of sentinel lymph node (SLN) in colorectal cancer.

Study, Year	Disease, Stage	No. Cases	ICG Administration	Dose	Fluorescence Evaluation	Detection Rate	Sensitivity (Metastasis in SL/Total Lymph Node Metastasis)
Carrara, A.; et al. [40], 2020	Colon cancer stage I-II, High risk colic polyposis	95	Laparoscopic/extraperitoneally	5 mL (5 mg/mL), sup + inf	Spies-Cam, Karl Storz; Firefly, da Vinci; PDE, Hamamatsu-Photonics	96.8%	85.9%
Hirsche, C.; et al. [41], 2012	Colon cancer, T1-3 N0	26	Laparoscopic	1–4 mL (5mg/mL) sup + inf	IC-View, Pulsion Medical Systems	96%	82%
Curie, A.; et al. [42], 2017	Colon cancer T1-2, N0	30	Endoscopic	1mL in 4 quadrants (5 mg/mL)	NIR Imaging System, Olympus	90%	33%
Liberale, G.; et al. [43], 2015	Colon cancer, Tis-T4, N0-2	2	Intraoperatively detected after 15 min + ex vivo	0.25 mg/kc, i.v.	PDE, Hamamatsu Photonics	N/A	N/A
Liberale, G.; et al. [44], 2016	Colon cancer, Tis-T4, N0-2	20	Ex vivo	0.5 mL in four sectors (0.5 mg/mL)	PDE, Hamamatsu Photonics	95%	43%
Watanabe, J.; et al. [45], 2016	Splenic flexure cancer stage I–II	31	Laparoscopic	1 mL (2.5 mg/mL) in two points	D-Light P, Karl Storz	100%	66%
Andersen, H.S.; et al. [46], 2017	Colic cancer T1-3, all cancers suited for laparoscopic surgery	29	Endoscopic	0.5 mL (25 mg ICG + 9 mL sterile water + 1 mL 20% human albumin) inf + sup	SPIES ICG camera with ICG Xenon 300 light source from Karl Storz	65.5%	20%
Nagata, K.; et al. [47], 2006	Colon/colorectal junction cancer T1-3, N0-2, all cancers suited for laparoscopic surgery	48	Subserosal	5 mL (5 mg/mL)	Laparoscope, Olympus	98%	53.6%
Kusano, M.; et al. [48], 2008	Colorectal cancer	26	Submucosal	2 mL (5 mg/mL)	PDE, Hamamatsu Photonics	88.5%	33.3%
Cahill, R.A.; et al. [49], 2012	Colon cancer stages I–III	18	Submucosal	2–3 mL	Laparoscope, Olympus	94%	100%
Van der Pass, M.H.G.M.; et al. [50], 2013	Colon cancer stages I–III	14	Subserosal	1 mL (2.5 mg/mL, containing 2% human serum albumin)	Laparoscope, Olympus	100%	0%
Nishigor, N.; et al. [35], 2016	Colorectal cancer T1-4 N0-2bM0	21	Submucosal/subserously	0.2–0.3 mL (2.5 mg/mL)	D-Light P System/Image 1 SPIES, Karl Stortz; laparoscope, Olympus	69.7%	45%
